# HLA polymorphism in neuroimmune diseases: linking antigen presentation to neuroinflammatory programs

**DOI:** 10.3389/fimmu.2026.1863697

**Published:** 2026-08-18

**Authors:** Shuo Liu, Siwen Yang, Jing Hu, Tao Bai, Jian-Ping Li

**Affiliations:** 1 The Fourth People’s Hospital of Shenyang, Shenyang, China; 2 Department of Science, Yokohama City University, Yokohama, Japan; 3 Department of Neurology, Shengjing Hospital of China Medical University, Shenyang, China; 4 HLA Laboratory, Liaoning Blood Center, Shenyang, China

**Keywords:** antigen presentation, glial activation, HLA polymorphism, immunogenetics, molecular mimicry, neuroimmune diseases, neuroinflammation

## Abstract

Human leukocyte antigen (HLA) polymorphism shapes antigen presentation and susceptibility to immune-mediated disease. In neuroimmune disorders, a central question is how HLA-dependent antigen visibility becomes disease-specific immunity and tissue injury. In this Review, we integrate genetic, molecular, cellular, and clinical evidence across multiple sclerosis (MS), neuromyelitis optica spectrum disorder, Guillain–Barré syndrome, narcolepsy, autoimmune encephalitis, and myasthenia gravis. The HLA-DR15-MS axis is the most mechanistically resolved example, linking allele-specific peptide display to autoreactive T-cell repertoires and convergent Epstein–Barr virus-related immune pathways. In other disorders, this upstream principle operates through distinct effectors, target tissues, molecular subtypes, and ancestral contexts, while causal peptide-receptor complexes often remain unresolved. We therefore separate association strength from mechanistic resolution and position glial activation and tissue injury mainly as downstream, context-dependent processes. This evidence-bounded synthesis identifies where HLA mechanisms are established, suggestive, or still inferred from association.

## 1 Introduction

Human leukocyte antigen (HLA) polymorphism is one of the most important genetic determinants of interindividual variation in immune recognition. By shaping antigen presentation, self–non-self discrimination, and the calibration of adaptive immune responses, HLA variation exerts a major influence on susceptibility to immune-mediated disease (1, 2). In neuroimmune diseases, this influence extends beyond simple genetic association. HLA polymorphism can influence which neural antigens become visible to the immune system, which autoreactive repertoires are selected, and how inflammatory responses are directed toward neural tissues (3–5).

Previous studies have established robust associations between specific HLA alleles and a wide range of neuroimmune diseases, including multiple sclerosis (MS), neuromyelitis optica spectrum disorder (NMOSD), narcolepsy, myasthenia gravis (MG), Guillain–Barré syndrome (GBS), chronic inflammatory demyelinating polyradiculoneuropathy (CIDP), and autoimmune encephalitis (AE) (6–12). These associations have traditionally been interpreted in terms of disease susceptibility, population genetics, or antigen-specific immune recognition. Although these perspectives remain important, they do not fully account for how HLA variation is translated into distinct inflammatory phenotypes across the central and peripheral nervous systems. HLA-dependent peptide presentation can influence tolerance, infection-triggered immune priming, and helper or cytotoxic effector programs. Autoantibody circuits and resident glial responses then depend on the disease-specific cellular and tissue context (4, 5, 13, 14).

A persistent gap in the literature is the limited integration of HLA association data with disease-specific neuroimmune mechanisms. Existing studies are often organized around one disease, allele, or immune pathway, leaving unclear which principles can be compared across disorders and which remain context specific. We address this gap through a single organizing question: how does HLA-dependent antigen visibility become a particular neuroinflammatory program? This question connects genetic association to peptide recognition, effector bias, target-tissue vulnerability, and clinical heterogeneity without assuming that every disease follows the same causal chain (5, 9, 13–15).

In this Review, we first define the structural, haplotypic, regulatory, and processing features that determine HLA-dependent antigen visibility (16–19). We then follow its translation through peptide-HLA-TCR recognition, tolerance, infection-related cross-reactivity, effector activation, and downstream tissue amplification (4, 5, 13, 14). The DR15-MS axis serves as the most mechanistically resolved anchor for this sequence, not as a model assumed to apply uniformly across neuroimmune diseases. We use the other disease contexts to identify where the sequence is supported, where it diverges, and where key links remain unresolved. This comparison then informs the translational opportunities and limits of HLA-based stratification.

## 2 HLA variation and functional architecture

### 2.1 Structural polymorphism and peptide-binding specificity

HLA-associated neuroimmune risk begins with variation in the peptide-binding groove. Polymorphic residues are concentrated around the pockets that anchor peptide side chains and shape the surface presented to T-cell receptors (TCRs) (17, 20). Consequently, two alleles can sample different peptide repertoires, bind the same peptide in different registers, or alter TCR-facing residues without changing the source protein. These effects define an allele-specific immunopeptidome rather than a generic capacity for antigen presentation (5).

HLA class I molecules present mainly intracellular peptides to CD8-positive T cells and also serve as ligands for killer-cell immunoglobulin-like receptors (KIRs). Class I variation can therefore affect both cytotoxic T-cell recognition and natural killer-cell inhibition or activation. These receptor interactions are established immunobiology, but their disease-specific contribution varies substantially across neuroimmune disorders (17, 21, 22).

HLA class II molecules present peptides derived largely from endosomal and extracellular proteins to CD4-positive T cells. Their functional consequences extend beyond helper-cell activation because CD4 T cells shape B-cell maturation, antibody production, regulatory networks, and inflammatory polarization. Class II associations are therefore especially prominent in antibody-mediated diseases and in MS, where the DR15 haplotype provides the best-developed mechanistic example (1, 5, 23).

The peptide-HLA-TCR complex is the relevant functional unit. Allele names alone do not identify the presented peptide, its binding register, the responding TCR, or the cellular context in which recognition occurs. Mechanistic claims should therefore specify these components whenever evidence permits and should distinguish structural binding evidence from patient-derived functional data.

This distinction sets the interpretive discipline used throughout the Review. A large odds ratio can identify a strong genetic marker without revealing the causal peptide or immune receptor, whereas a solved peptide-HLA-TCR structure can establish molecular plausibility without proving that the complex initiates disease in patients. We therefore compare association strength and mechanistic resolution as separate dimensions.

Throughout this Review, we use the term immunopeptidome for the complete set of peptides displayed by HLA molecules in a defined cell or tissue context. This concept connects HLA sequence variation to antigen visibility and provides a testable bridge between genetic association and immune recognition (5, 23).

### 2.2 Linkage disequilibrium, extended haplotypes, and ancestry

The major histocompatibility complex is gene dense and contains extensive linkage disequilibrium (LD). Disease-associated alleles are often inherited with other classical HLA alleles, regulatory variants, and non-HLA immune genes (16, 18, 25). An observed allele may therefore be causal, may modify another causal variant, or may mark an extended haplotype that cannot be resolved by standard association analysis.

The DR15 haplotype illustrates this problem: it encodes two linked HLA-DR heterodimers, conventionally termed DR2a (HLA-DRA*01:01 paired with HLA-DRB5*01:01) and DR2b (HLA-DRA*01:01 paired with HLA-DRB1*15:01), which present overlapping but distinct peptide repertoires (23). DRB1*15:01 is commonly inherited with DQA1*01:02, DQB1*06:02, and DRB5*01:01. High-resolution sequencing in European-American cohorts associated DRB1*15:01 with MS risk, but also showed that linked DRB5 and extended DR-DQ configurations contribute additional information (26). Mechanistic studies likewise indicate that DR2a and DR2b can jointly shape autoreactive T-cell repertoires (23).

Extended haplotypes can also produce subtype-specific or opposing effects. In MG, HLA-B*08 strongly marks early-onset disease, whereas class II signals differ in late-onset and muscle-specific kinase (MuSK)-positive disease (27–30). Treating MG as one phenotype would therefore obscure clinically relevant genetic heterogeneity.

Ancestry changes both allele frequency and LD structure. The pooled association between the DRB1*03 group and historical neuromyelitis optica (NMO) cohorts was OR 2.46, but the dominant signal differed between Western and East Asian populations (10). These differences preclude a universal effect estimate for all neuromyelitis optica spectrum disorder (NMOSD) populations.

Population stratification is therefore a biological and statistical issue. Ancestry-specific haplotypes can alter the apparent magnitude, direction, or independence of an HLA association. Cross-population comparisons should retain phenotype definition, serostatus, typing resolution, and haplotypic context rather than pooling incompatible estimates (10, 25, 31).

These constraints are especially important for rare diseases, where small cohorts can produce apparently large effects. Strong signals in anti-LGI1 encephalitis and MuSK-positive MG are informative, but the estimates remain sensitive to ancestry, phenotype definition, and LD (29, 32, 33).

Accordingly, **Table 1** reports representative, source-specific estimates rather than a single pooled HLA effect for each disease. The estimates should be read as evidence within defined populations and molecular subtypes, not as universal measures of genetic risk.

**Table 1:** Representative HLA associations across neuroimmune diseases.

Disease/subtype	HLA factor	Direction	Population/sample	Effect estimate	Mechanistic interpretation	Evidence grade	Key limitation/reference
Multiple sclerosis	DRB1*15:01 within DR15	Risk	European American; 1,403 cases and 1,425 controls	OR 3.20; p<2.2 x 10^-16	Human immunopeptidomic, structural, tissue, and T-cell evidence supports altered peptide display and autoreactive repertoire shaping.	Association B; mechanism M1-M2	Ancestry- and haplotype-dependent; LD limits single-allele attribution (23, 24, 26).
Multiple sclerosis	DR4-DQ haplotype	Protective	European American; DRB1*15:01-negative analysis	OR 0.64; p=0.028	Haplotype-dependent peptide presentation.	Association C; mechanism M4	Conditional, cohort-specific signal requiring replication (26).
Historical NMO cohorts	DRB1*03 group	Risk	Meta-analysis; 568 cases across Asian, Latin American, and European cohorts	OR 2.46 (95% CI 2.01-3.01)	Class II susceptibility in predominantly antibody-mediated disease.	Association A; mechanism M4	Older diagnostic criteria and incomplete AQP4-IgG stratification (10).
Early-onset AChR-positive MG	HLA-B*08	Risk	North European; 649 cases	OR 6.41; p=2.87 x 10^-113	Marks the extended 8.1 ancestral haplotype; class I causality is unresolved.	Association B; mechanism M4	Strong LD; does not alone prove a CD8 T-cell mechanism (27).
Late-onset AChR-positive MG	DRB1*07-DQB1*02	Risk	Italian; 107 generalized non-thymoma cases	OR 4.10 (95% CI 2.80-5.99)	Class II-dependent helper-cell support is plausible.	Association C; mechanism M4	Single ancestry and narrowly defined subtype (30).
Late-onset AChR-positive MG	DQA1*05:01	Protective	European; 532 cases and 2,128 controls	OR 0.54; p=5.9 x 10^-12	Subtype-dependent class II susceptibility.	Association B; mechanism M4	Direction differs from early-onset disease (28).
MuSK-positive MG	DQB1*05:02	Risk	Turkish subtype cohort	OR 8.56; p=6.88 x 10^-13	Class II predisposition to an IgG4-dominant autoimmune response.	Association C; mechanism M4	Rare subtype and single population (29).
Guillain-Barré syndrome	DQB1 allele groups	Inconsistent	Meta-analysis; 780 cases and 1,353 controls	DQB1*030x: OR 0.76 (0.57-1.03); DQB1*060x: OR 1.48 (0.96-2.29)	Ganglioside mimicry is stronger evidence than a universal HLA effect.	Association U/C; mechanism M4 for HLA	Trigger, subtype, ancestry, and small studies produce heterogeneity (49, 50).
Narcolepsy type 1	DQB1*06:02	Risk	Meta-analysis; 2,077 cases and 7,802 controls across four ethnic groups	OR 24.1 (95% CI 14.6-39.5)	HLA-selective susceptibility with patient-derived T-cell evidence against hypocretin-neuron antigens.	Association A; mechanism M2	Strong susceptibility is not absolute necessity; initiating trigger remains unresolved (54, 55).
Anti-LGI1 encephalitis	DRB1*07:01-DQA1*02:01-DQB1*02:02 region	Risk	European GWAS; 54 cases and 1,194 controls	Leading SNP OR 13.66 (95% CI 7.50-24.87)	Strong HLA class II-region predisposition to antibody-mediated encephalitis.	Association B; mechanism M4	OR is for rs2858870, not directly for the classical haplotype (32).
Anti-NMDAR encephalitis	DQB1*05:02	Risk	Chinese Han; 413 cases and 7,127 controls	OR 2.10 (95% CI 1.70-2.59)	Class II susceptibility; docking evidence remains computational.	Association B/C; mechanism M4	Single-ancestry GWAS; not a universal multiethnic signal (51, 53).
Anti-NMDAR encephalitis	HLA-B position 97R	Protective	Chinese Han; same GWAS	OR 0.63 (95% CI 0.53-0.74)	Class I peptide-groove variation may alter susceptibility.	Association B/C; mechanism M4	May tag linked variation; functional validation is absent (51).

Evidence grades are descriptive rather than validated scores. Association grades A, B, C, and U summarize replication and genetic resolution, whereas M1–M4 indicate the proximity of mechanistic evidence to patient disease. Effect estimates are study-, population-, and subtype-specific and should not be directly compared across rows or interpreted as measures of causal importance. Abbreviations: CI, confidence interval; GWAS, genome-wide association study; OR, odds ratio.

### 2.3 Regulatory variation and context-dependent HLA expression

Peptide-binding specificity is only one layer of HLA function. Promoters, enhancers, expression quantitative trait loci, epigenetic state, transcript processing, and allele-specific expression can alter the abundance and timing of HLA display (19). These regulatory effects can influence whether a potentially relevant peptide-HLA complex is expressed at a meaningful level in disease-relevant cells and tissues.

Interferon-rich inflammation can induce HLA class I and class II expression in infiltrating antigen-presenting cells and resident neural cells (34–37). This induction may broaden local antigen visibility after immune activation. However, evidence that specific regulatory variants directly drive expression in human neural tissue remains limited for many neuroimmune diseases.

Regulatory associations should therefore be interpreted in their measured cellular context. An eQTL detected in blood does not automatically establish the same effect in microglia, astrocytes, thymic epithelial cells, or disease lesions. Tissue-resolved expression and immunopeptidomic studies are needed before such signals are incorporated into disease-specific causal chains.

This boundary is important for glial biology. Microglia and astrocytes can express antigen-presentation machinery under inflammatory conditions, but their role is usually downstream of peripheral immune priming. Local HLA expression may amplify an established response without being the initiating event (14, 37).

### 2.4 Antigen-processing partners and immune-receptor interactions

ERAP1 and ERAP2 trim HLA class I ligands and can reshape the class I immunopeptidome. Robust ERAP-HLA epistasis is established in several class I-associated inflammatory diseases, but comparable genetic or functional evidence in MS remains limited (5). ERAP1/2 should therefore be presented as context-dependent candidate modifiers rather than components of an established MS pathway.

KIR-HLA interactions provide a second class I module. MS studies have reported associations with KIR genes or HLA ligands, including a protective KIR2DS1 signal in one Portuguese cohort, but loci and directions have not been consistently replicated (21, 22). KIR-HLA biology is therefore established at the receptor level, while its disease-specific contribution to MS remains provisional.

To make such differences transparent, **Tables 1** and **2** use a descriptive two-axis evidence framework rather than a validated scoring instrument. Association grades A, B, C, and U denote decreasing replication or resolution of genetic evidence. Mechanistic evidence-proximity labels M1-M4 distinguish direct patient-derived evidence, human structural or ex vivo evidence, disease-model evidence, and general immunobiology or cross-disease analogy. The axes remain independent and are used to guide interpretation, not to calculate a composite certainty score.

**Table 2:** Comparative strength of HLA association and mechanistic evidence proximity.

Disease context	Association evidence	Mechanistic evidence proximity	Most defensible interpretation
Multiple sclerosis/DR15	A	M1-M2	Strongest integrated link from haplotype and peptide display to patient-derived T-cell biology; causal sufficiency remains unproven.
Narcolepsy type 1	A	M2	Large DQB1*06:02 effect and patient T-cell evidence; initiating trigger and universal peptide-HLA-TCR route remain unresolved.
Anti-LGI1 encephalitis	B	M4	Strong HLA class II-region predisposition; no validated initiating LGI1 peptide-HLA complex.
Myasthenia gravis	B-C by subtype	M4	HLA effects depend on antibody subtype, age at onset, thymoma status, ancestry, and extended haplotype.
NMOSD/historical NMO	A-B	M4	Replicated but ancestry- and phenotype-dependent class II associations; direct peptide mechanism remains limited.
Anti-NMDAR encephalitis	B-C	M4	Class I and II signals vary across ancestries and clinical contexts; current functional evidence is indirect or computational.
Guillain–Barré syndrome	U-C	M4 for HLA	Campylobacter-ganglioside mimicry is better established than a universal HLA contribution.

Association grades A, B, C, and U summarize replication and genetic resolution; M1–M4 indicate the proximity of mechanistic evidence to patient disease. These categories are descriptive and do not constitute a validated scoring system.

## 3 Mechanistic bridge from HLA variation to neuroimmunity

### 3.1 Immunopeptidome and peptide-HLA-TCR recognition

A mechanistic interpretation of HLA risk requires evidence that connects an allele to a peptide repertoire, an immune receptor, and a disease-relevant cell or tissue. The strongest current example is the DR15 haplotype in MS. Wang and colleagues characterized DR2a and DR2b immunopeptidomes in primary human cells, thymus, and MS brain tissue, and identified autoreactive CD4 T-cell clones that cross-recognized HLA-DR-derived self-peptides, microbial peptides, and neural autoantigens (23).

This work supports a model in which DR15 molecules function both as peptide-presenting structures and as sources of self-peptides. It also shows why DRB1*15:01 should not be discussed in isolation from DRB5*01:01. The two allomorphs can present distinct but overlapping peptide repertoires and jointly shape the available autoreactive CD4+ T-cell repertoire (23).

Myelin basic protein (MBP) provides a well-developed structural example. MBP85–99 and related registers have been visualized in HLA-DR2 complexes in MS lesions, and structural studies show how DR2a and DR2b alter peptide register and TCR engagement (38–40). This evidence supports a mechanistic basis for allele-dependent recognition, but it does not identify MBP85–99 as the sole or universal initiating antigen in MS.

By contrast, much of the evidence for MOG35–55 in a DRB1*15:01 context comes from HLA-transgenic experimental autoimmune encephalomyelitis models (41, 42). MOG35–55 is therefore useful for testing allele-restricted biology *in vivo*, but it should be labelled as model-based evidence rather than an established dominant human MS epitope.

These examples define the appropriate evidentiary ladder. Patient-derived tissue and functional data support disease-context mechanisms; structural and ex vivo binding data support molecular plausibility; transgenic models test biological consequences; and sequence prediction alone remains hypothesis-generating.

### 3.2 Central and peripheral tolerance

HLA variation can shape autoreactivity before clinical disease by altering the self-peptides displayed during thymic selection (4, 43). Incomplete or atypical presentation may allow potentially autoreactive clones to survive, whereas presentation under tolerogenic conditions can promote deletion or regulatory lineages. These outcomes depend on peptide abundance, presenting cell type, and TCR affinity rather than on allele identity alone.

The DR15 immunopeptidome study provides human evidence relevant to this model. HLA-DR-derived self-peptides were presented in thymus and recognized by autoreactive T-cell clones, linking HLA expression to repertoire formation (23). The study supports a route through which two linked DR15 allomorphs may shape cross-reactive repertoires, but it does not prove that this route is sufficient to cause MS.

Peripheral tolerance adds further checkpoints. Regulatory T cells, anergy, co-inhibitory pathways, and the anatomical context of antigen presentation can restrain autoreactive cells that escape central deletion (13, 44). Infection, inflammation, or tissue injury may overcome these controls by increasing antigen availability and co-stimulation.

Tolerance failure should therefore be described as conditional. A susceptible HLA background can create a permissive repertoire, but disease expression may additionally depend on environmental exposure, immune activation, and access to the relevant target tissue. This boundary is particularly important when moving from structural binding studies to causal claims.

The same principle applies across diseases. Strong HLA associations in narcolepsy or anti-LGI1 encephalitis identify selective immune predisposition, but they do not reveal a complete sequence from thymic selection to tissue injury. Mechanistic depth remains disease specific.

### 3.3 Infection, molecular mimicry, and cross-reactivity

Infection can convert an HLA-shaped repertoire into active autoimmunity when microbial and self-antigens are cross-recognized. In MS, Epstein-Barr virus (EBV) provides the most developed example, but at least two mechanistically distinct routes should be separated.

Lanz and colleagues identified clonally expanded cerebrospinal-fluid B cells and antibodies that recognized both EBV nuclear antigen 1 (EBNA1) and the glial adhesion molecule GlialCAM (45). Patient-derived antibodies, structural analysis, and model experiments support antibody molecular mimicry. This study does not, by itself, prove a DRB1*15:01-restricted T-cell mechanism.

Wang and colleagues identified a separate T-cell route in which DR15-restricted autoreactive CD4 T-cell clones cross-recognized HLA-DR self-peptides, EBV peptides, Akkermansia peptides, and autoantigens (23). More recent work linked EBV infection directly to HLA-DR15-dependent myelin antigen presentation by showing that EBV-infected HLA-DR15-positive B cells present MBP peptides overlapping with peptides detected in MS brain tissue, and that CD4-positive T cells from HLA-DR15-positive patients with MS can recognize these MBP peptides (24). Together, these studies support convergent EBV-related antibody, infected B-cell antigen-presentation, and T-cell mechanisms, while leaving their relative contribution to disease initiation unresolved.

Molecular similarity alone is insufficient evidence of pathogenic mimicry. A convincing claim requires HLA binding, receptor cross-recognition, disease-relevant immune cells, and functional consequences. This standard prevents sequence resemblance or computational docking from being presented as an established disease mechanism.

GBS illustrates a different relationship between infection and autoimmunity. Campylobacter jejuni glycans can induce antibodies that cross-react with neural gangliosides, providing a strong infection-to-target mechanism (46–48). However, HLA association evidence in GBS is considerably less consistent, so the ganglioside mimicry mechanism should not be described as uniformly HLA-dependent (49, 50).

### 3.4 Effector pathways: helper T cells, cytotoxic cells, B cells, and NK cells

Peptide-HLA recognition initiates downstream programs whose relative importance depends on disease context. Class II-restricted CD4 T cells can provide cytokines and B-cell help, whereas class I-restricted CD8 T cells can recognize infected or stressed cells. HLA class I also regulates NK-cell responses through KIR ligands (1, 5, 17).

These pathways should not be assigned equal weight in every disease. Helper-cell and B-cell interactions are central to autoantibody-mediated MG and autoimmune encephalitis, but the disease-associated HLA alleles differ by antibody subtype and age at onset (27–30, 32, 51). In MS, CD4 T-cell evidence is strongest for DR15, whereas KIR-HLA associations remain provisional (21–23).

Class I associations can nevertheless reveal relevant biology. HLA-B*08 marks early-onset MG in European cohorts, but its strong LD within the 8.1 ancestral haplotype limits attribution to one gene or to CD8 T cells alone (27). Similar caution applies to protective class I amino-acid signals in anti-NMDAR encephalitis (51).

B-cell and antibody responses also differ mechanistically. IgG1/IgG3 antibodies can activate complement, whereas MuSK-positive MG is often dominated by IgG4 antibodies that disrupt protein interactions without efficient complement activation. HLA association therefore identifies upstream immune selection, not a uniform downstream effector pathway.

**Figure 1:**
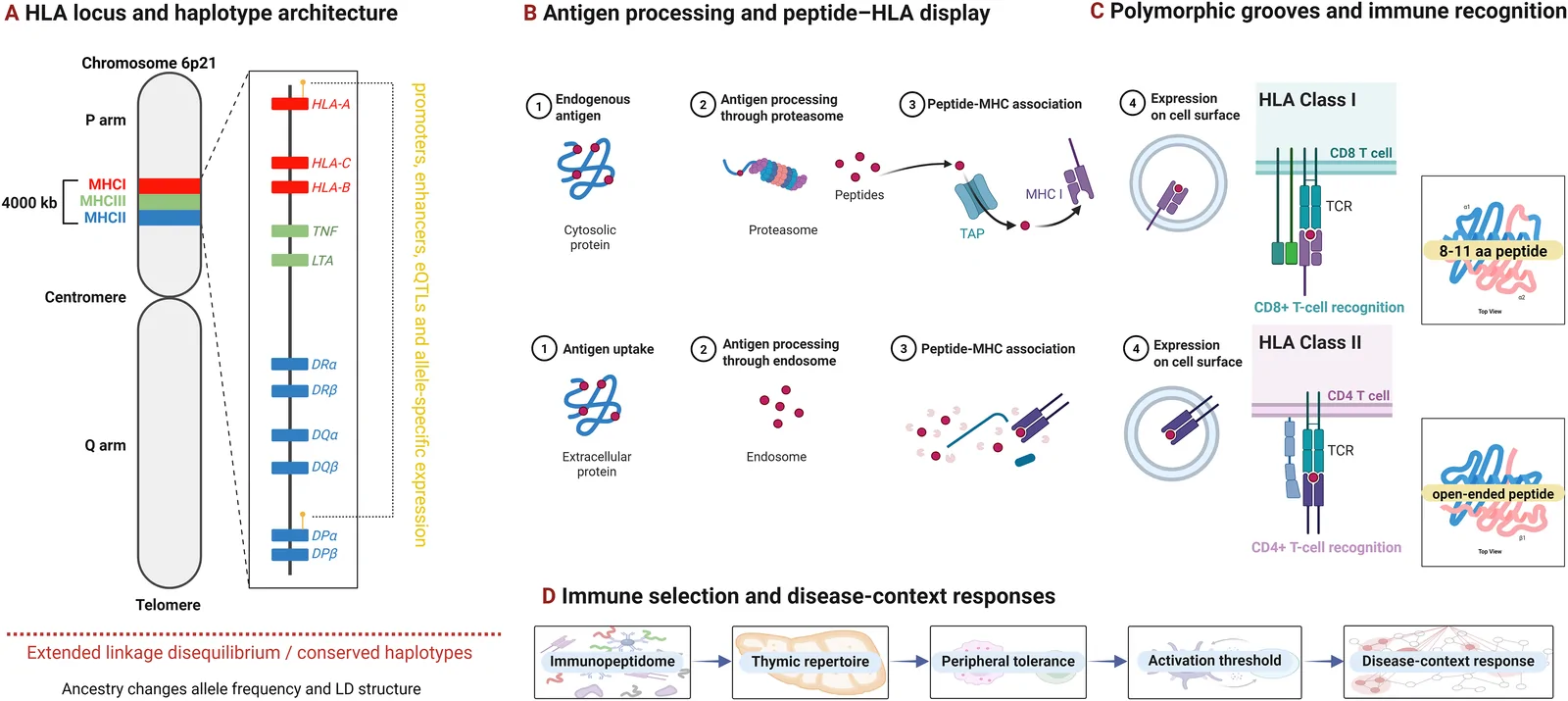
Functional architecture of HLA variation shaping antigen visibility. **(A)** HLA locus and haplotype architecture, including class I, class III and class II regions, extended linkage disequilibrium and regulatory variation. **(B)** Antigen-processing routes converge on peptide-HLA display through class I and class II pathways. **(C)** Polymorphic peptide-binding grooves shape peptide length, register and immune-receptor recognition. **(D)** These layers influence the immunopeptidome, immune repertoire selection, tolerance, activation threshold and disease-context responses. Arrows denote functional relationships and do not imply a universal disease-causal sequence.

The most defensible synthesis is that HLA variation weights an immune network. It does not switch a single pathway on or off. Disease phenotype emerges from the combination of antigen presentation, receptor repertoire, co-stimulation, antibody class, and tissue-specific vulnerability. The upstream architecture linking HLA variation, antigen processing, peptide display, and immune recognition is summarized in **Figure 1**.

**Figure 2:**
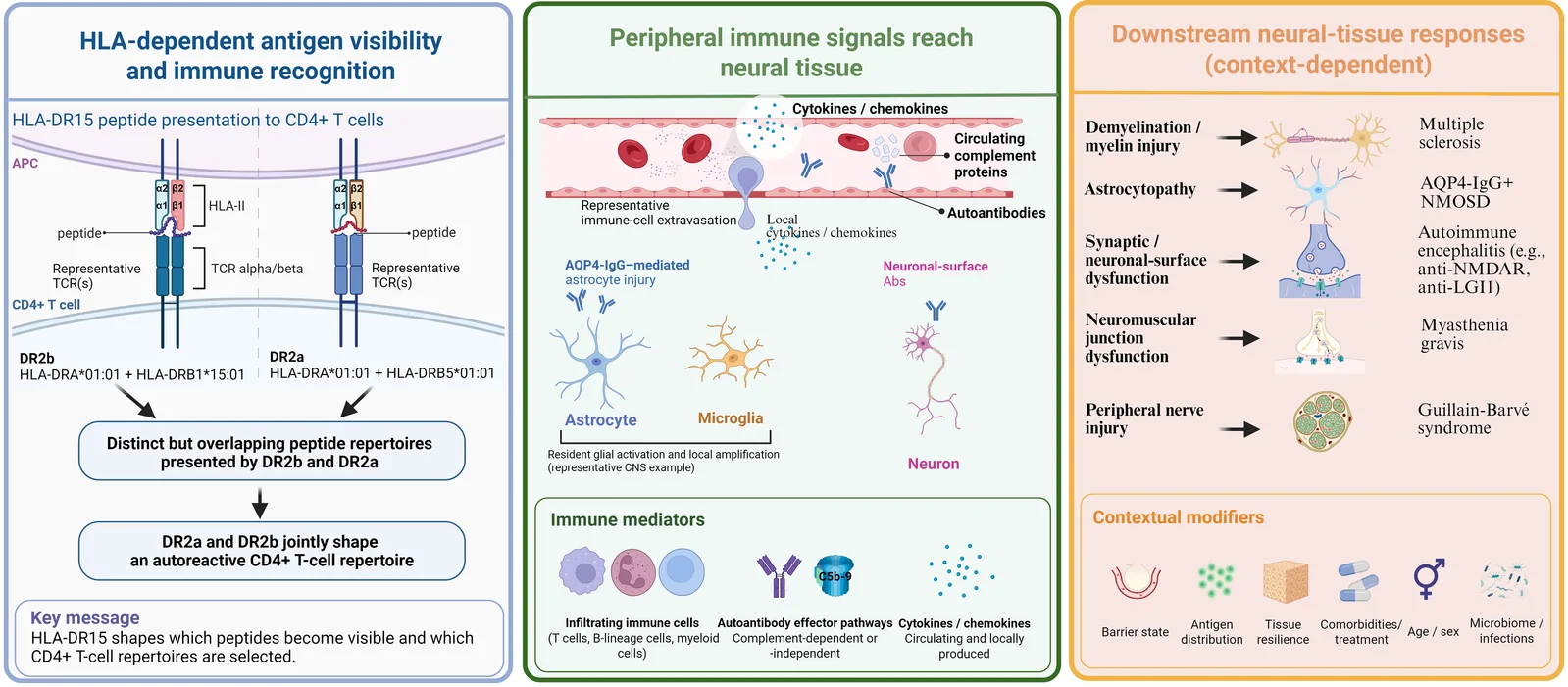
HLA-dependent antigen visibility and context-dependent neuroimmune responses. The left panel uses HLA-DR15 in multiple sclerosis as the mechanistic anchor. DR2b (HLA-DRA*01:01 + HLA-DRB1*15:01) and DR2a (HLA-DRA*01:01 + HLA-DRB5*01:01) present distinct but overlapping peptide repertoires and jointly shape an autoreactive CD4+ T-cell repertoire. The middle panel illustrates how infiltrating immune cells, autoantibody effector pathways, and circulating or locally produced cytokines and chemokines connect peripheral immunity with neural tissue. AQP4-IgG-mediated astrocyte injury, neuronal-surface antibodies, and resident glial activation are shown as representative, context-dependent mechanisms. The right panel summarizes downstream neural-tissue responses and the contextual modifiers that influence their expression. This evidence-bounded schematic does not imply that every link is established or shared across all disorders.

This network view defines the role of **Figure 2**: the schematic summarizes how HLA-shaped antigen visibility and peripheral immune mediators feed into context-dependent neural tissue responses, while **Tables 1** and **2** retain the evidence boundaries for association strength and mechanistic support.

Separating the pathway schematic from evidence grading is important because association magnitude does not measure mechanistic certainty. A large genetic effect can coexist with an unresolved antigen, whereas a detailed molecular structure can describe one interaction without establishing its population-level importance.

### 3.5 Downstream glial and tissue amplification

Glial activation is best positioned downstream of peripheral immune priming. Infiltrating lymphocytes, antibodies, complement, and inflammatory cytokines can activate microglia and astrocytes, which then amplify local injury through cytokine release, altered barrier function, phagocytosis, and metabolic stress (3, 14, 37).

Microglia can upregulate HLA class II and process antigens in inflammatory environments. This capacity may sustain local T-cell interactions in MS lesions, but it does not establish microglia as the initial site of tolerance failure. The initiating antigen-presenting cell and anatomical compartment remain uncertain in many disease settings.

Astrocytes occupy a different position. In NMOSD, they are primary targets of AQP4-IgG-mediated injury, whereas in MS they more often participate in lesion containment, barrier regulation, and inflammatory amplification (10, 37, 52). The same term, glial activation, therefore describes biologically distinct roles.

Peripheral nerve disease requires another tissue model. In GBS, complement, macrophages, antibodies, and autoreactive T cells act at peripheral nerves and roots rather than through a CNS glial program (46). A universal glia-centered pathway would therefore obscure disease-specific anatomy.

Local amplification also differs temporally. It may contribute to chronic lesion evolution in MS, acute astrocyte injury in NMOSD, synaptic dysfunction in autoimmune encephalitis, or secondary inflammation after antibody binding. These processes are downstream consequences of distinct upstream immune routes.

Taken together, these mechanisms define a common sequence without implying a universal pathway: HLA variation first alters antigen visibility and immune-receptor engagement; disease context then determines the dominant effector route, anatomical access, and local tissue response. This ordering prevents downstream inflammation from being mistaken for an allele-specific initiating mechanism.

The disease sections below therefore ask the same questions in each context: how strong is the HLA association, which biological link is directly supported, and where does the mechanistic chain remain inferred? Direct patient evidence supports parts of the DR15-MS and EBV-MS sequence, whereas many glial and tissue branches still rely on convergent pathology rather than allele-specific experiments.

## 4 Disease-context evidence

### 4.1 Demyelinating neuroimmunity: MS as the mechanistic anchor and NMOSD as a contrasting context

MS provides the strongest bridge from HLA association to allele-specific mechanism. DRB1*15:01 within the DR15 haplotype is the major class II risk allele in European-ancestry populations. A high-resolution European-American study reported OR 3.20 for DRB1*15:01:01:01SG, while linked DRB5 and extended DR-DQ haplotypes also contributed (26). The estimate is representative rather than universal because ancestry and haplotypic models alter effect size.

Mechanistic evidence converges at the peptide-HLA-TCR interface. DR2a and DR2b immunopeptidomes from human immune cells, thymus, and MS brain tissue support joint shaping of autoreactive CD4 T-cell repertoires (23). MBP85–99 provides a human structural and lesion-associated example, whereas MOG35–55 remains primarily an HLA-transgenic model antigen (38–42).

EBV adds an environmental layer. EBNA1-GlialCAM antibody cross-reactivity, DR15-restricted T-cell cross-recognition, and EBV-infected HLA-DR15-positive B-cell presentation of MBP peptides provide distinct but complementary routes (23, 24, 45). No single route alone establishes a sufficient cause of MS. Their importance lies in demonstrating how infection can engage an HLA-shaped autoreactive repertoire.

Protective HLA effects further show that the locus does not act through one risk allele. In DRB1*15:01-negative European-American analyses, one DR4-DQ haplotype was protective (OR 0.64), whereas another closely related DR4-DQ haplotype increased susceptibility (26). Peptide-binding context and LD are therefore more informative than broad serological groups.

NMOSD should not be forced into the MS mechanism. A meta-analysis of historical NMO cohorts associated the DRB1*03 group with disease (OR 2.46, 95% CI 2.01-3.01), particularly in Western populations, while DPB1*05:01 was prominent in East Asian cohorts (10). Current AQP4-IgG-positive definitions were not uniformly applied across those studies.

The dominant NMOSD effector mechanism is AQP4-IgG-mediated astrocyte injury, followed by complement activation and inflammatory recruitment. HLA class II associations may support upstream helper-cell and antibody responses, but a defined allele-AQP4 peptide-TCR pathway is less developed than the DR15-MS mechanism.

MS and NMOSD therefore illustrate how the shared upstream question can lead to different evidence architectures. MS combines replicated association with human immunopeptidomic, structural, cellular, and tissue evidence. NMOSD combines ancestry-dependent HLA associations with a well-defined pathogenic antibody and astrocyte target, while the intervening HLA-restricted peptide-receptor mechanism remains less resolved. The contrast preserves a common framework without forcing NMOSD into an MS-like pathway.

### 4.2 Guillain-Barré syndrome: strong mimicry, inconsistent HLA association

GBS is best understood through the interaction between infection, microbial glycans, ganglioside cross-reactivity, and peripheral nerve injury. Campylobacter jejuni is the best-characterized trigger, although other infections can precede disease (46–48). This mechanism is more firmly supported than any universal HLA association.

A meta-analysis of nine HLA-DQB1 studies, including 780 cases and 1,353 controls, found no significant overall association. DQB1*030x in Asian cohorts showed OR 0.76 (95% CI 0.57-1.03), while DQB1*060x overall showed OR 1.48 (95% CI 0.96-2.29) (49). Neither estimate supports a stable generic risk allele.

A broader field synopsis reported associations with selected DRB1 alleles, including DRB1*04:01 and DRB1*13:01, but results varied by ancestry and subtype (50). The limited number of independent studies and differences between acute inflammatory demyelinating polyneuropathy and acute motor axonal neuropathy reduce confidence in pooled mechanistic interpretation.

The most appropriate conclusion is therefore asymmetric: infection-triggered ganglioside mimicry is an established disease mechanism, whereas HLA-dependent susceptibility is suggestive and heterogeneous. HLA may modify antigen processing or helper-cell support in subsets, but current evidence does not justify drawing one allele-specific causal chain for GBS.

This distinction also prevents overgeneralization from newly identified autoreactive peripheral-nerve T cells (46). Such cells strengthen evidence for adaptive immunity in GBS, but their presence does not establish a common HLA restriction across all triggers and clinical variants.

Related chronic inflammatory neuropathies should be discussed separately. Their triggers, temporal course, and HLA signals differ from acute GBS, and current evidence does not support treating them as one immunogenetic spectrum.

GBS consequently serves as an instructive counterexample in this Review: a well-supported antigenic mimicry mechanism can coexist with comparatively weak and inconsistent HLA association evidence.

### 4.3 Autoimmune encephalitis and myasthenia gravis: subtype-specific HLA effects

Autoimmune encephalitis is immunogenetically heterogeneous. In anti-LGI1 encephalitis, a European genome-wide association study identified a strong HLA class II-region signal. The leading SNP had OR 13.66 (95% CI 7.50-24.87), and imputation implicated the DRB1*07:01-DQA1*02:01-DQB1*02:02 haplotype (32). The SNP estimate should not be reported as a haplotype-specific OR.

A small Korean cohort provided ancestry-specific corroboration, with 10 of 11 patients carrying DRB1*07:01-DQB1*02:02 (33). The frequency is striking but the sample is too small for a universal effect estimate. Together, the studies support strong HLA class II predisposition without yet identifying a validated LGI1-derived initiating peptide.

Anti-NMDAR encephalitis shows a different pattern. In 413 Chinese Han cases and 7,127 controls, DQB1*05:02 was associated with risk (OR 2.10, 95% CI 1.70-2.59), whereas HLA-B position 97R was protective (OR 0.63, 95% CI 0.53-0.74) (51). Computational docking is hypothesis-generating and does not constitute direct peptide-presentation evidence.

A multiethnic study instead reported weaker DRB1*01:01-DQA1*01:01-DQB1*05:01 and DRB1*11:01 signals, particularly in European descendants and patients without teratoma (53). Anti-NMDAR encephalitis therefore does not yet have one classical allele that is consistently replicated across ancestries and clinical contexts.

MG requires equally strict subtype separation. HLA-B*08 was strongly associated with early-onset AChR-positive disease in North Europeans (OR 6.41), but LD within the 8.1 ancestral haplotype limits causal assignment (27). In late-onset AChR-positive disease, an Italian study associated DRB1*07-DQB1*02 with risk (OR 4.10, 95% CI 2.80-5.99) (30).

Late-onset disease also illustrates protective effects: DQA1*05:01 showed OR 0.54 in one European analysis, opposite to its direction in early-onset disease (28). In Turkish MuSK-positive MG, DQB1*05:02 showed OR 8.56, with additional support for DRB1*14, DRB1*16, and DQB1*05 (29). These findings should not be generalized to AChR-positive MG.

AE and MG therefore support a shared principle rather than a shared allele. Class II variation can select helper-cell and B-cell responses against highly specific surface antigens, but effect size and downstream antibody biology depend on molecular subtype, ancestry, age at onset, tumor status, and thymic context.

### 4.4 Narcolepsy as a strong HLA-selective neuroimmune phenotype

Narcolepsy type 1 (NT1) has one of the strongest HLA associations in neuroimmunology. A systematic meta-analysis of 12 studies included 2,077 NT1 cases and 7,802 controls across four major ethnic groups. DQB1*06:02 conferred pooled adjusted OR 24.1 (95% CI 14.6-39.5) (54).

The same allele had a smaller association with narcolepsy type 2 (OR 3.9, 95% CI 2.2-6.8) (54). Combining the two phenotypes would therefore dilute both clinical and biological interpretation. DQB1*06:02 is highly informative for NT1 susceptibility, but its absence does not absolutely exclude disease.

Human T-cell studies provide mechanistic support for autoreactivity against antigens expressed by hypocretin neurons (55). These findings connect strong HLA susceptibility with a restricted neuronal target, although the initiating environmental trigger and the dominant pathogenic T-cell population remain under investigation.

Narcolepsy therefore differs from disorders with widespread inflammatory lesions. Its pathogenic importance lies in selective immune injury to a small neuronal population rather than in the volume of inflammation. This makes NT1 a useful example of HLA-dependent target precision.

The evidence remains graded rather than absolute. The genetic association is strong and replicated, and patient-derived T-cell evidence supports biological plausibility. However, no single peptide-HLA-TCR pathway has been shown to account for all cases.

Across these disease contexts, three patterns recur. Effect size and mechanistic resolution do not move in parallel; the same clinical label can contain genetically distinct molecular subtypes; and a shared upstream role for antigen selection can lead to different effector and tissue outcomes. NT1 and anti-LGI1 encephalitis have large HLA-region effects, whereas MS currently offers the most detailed multilayered mechanism.

**Table 1** preserves the quantitative strength of this evidence by reporting representative associations, confidence intervals, populations, evidence grades, and limitations. These estimates calibrate the disease narratives; they are not a ranking of biological importance across incompatible study designs.

## 5 Discussion: cross-disease synthesis, shared logic, unequal evidence

### 5.1 Antigen visibility is the common upstream principle

Across neuroimmune diseases, HLA variation most consistently acts upstream by changing which self- or pathogen-derived peptides become visible to adaptive immunity. This antigen-visibility principle organizes the cross-disease comparison: peptide selection shapes receptor engagement and effector bias, while barrier access, antigen distribution, target-cell biology, and local inflammatory responses determine where and how injury appears. These are recurring axes of neuroinflammatory organization rather than a universal causal pathway. Their disease-specific resolution is greatest in MS, where the DR15 haplotype can be connected to defined immunopeptidomes, peptide-HLA complexes, and patient-derived autoreactive T-cell responses (23, 38–40).

Large genetic effects do not necessarily imply equivalent mechanistic depth. DQB1*06:02 in narcolepsy type 1 and the HLA class II region in anti-LGI1 encephalitis show strong associations, yet the initiating peptide-HLA-TCR sequence remains incomplete (32, 33, 54, 55). Conversely, GBS has a well-supported Campylobacter-ganglioside mimicry mechanism but lacks a consistent universal HLA association (46–50).

The cross-disease conclusion is therefore limited but useful: HLA-dependent antigen selection is a recurrent entry point, whereas the allele, peptide, responding receptor, and causal contribution must be established separately in each molecular subtype and ancestral context.

### 5.2 Effector pathways are disease-context outputs

After peptide-HLA recognition, the dominant effector route depends on the target antigen and tissue context. Class II-biased responses can support CD4 T-cell activation, B-cell maturation, and pathogenic antibodies, as illustrated by antibody-defined encephalitis and MG. Class I pathways may recruit CD8 T cells or modify NK-cell signaling, but disease-specific evidence for KIR-HLA causality remains provisional (7, 9, 21, 22, 51, 53, 56, 57).

These pathways should not be collapsed into rigid labels such as T-cell-mediated or antibody-mediated disease. MS combines T-cell, B-cell, and innate amplification; MG subtypes differ in antibody class and HLA architecture; and anti-NMDAR encephalitis shows ancestry-dependent class I and class II signals (23, 27–30, 45, 51, 53). HLA therefore biases an immune response rather than uniquely determining its final form.

This distinction also prevents odds ratios from being treated as mechanistic rankings. Association magnitude identifies genetic enrichment within a defined study population; it does not specify the pathogenic cell, peptide, or receptor.

### 5.3 Glial engagement and tissue injury are mainly downstream

Resident neural cells shape the local consequences of an HLA-shaped adaptive response. Microglia and astrocytes respond to infiltrating lymphocytes, antibodies, cytokines, barrier disruption, and tissue damage. Under inflammatory conditions they may also express antigen-presentation machinery, but current evidence more strongly supports their role as downstream effectors and amplifiers than as universal initiating cells (14, 37).

Tissue accessibility and vulnerability further constrain disease expression. The same upstream immune predisposition cannot explain why one response targets myelin, another targets a neuronal surface receptor, and another produces selective hypocretin-neuron loss. Barrier properties, antigen distribution, target-cell biology, and repair capacity act as downstream filters between immune recognition and clinical phenotype (3, 9, 37, 52).

Accordingly, glial engagement and tissue vulnerability should be interpreted as contextual modifiers of an ongoing immune response. Direct attribution of these outcomes to an HLA allele requires longitudinal or experimental evidence that is still absent for most disorders.

### 5.4 Central and peripheral neuroinflammatory programs

HLA polymorphism can contribute to both central and peripheral neuroinflammatory programs, but the anatomical compartment changes how antigen visibility is translated into disease. In CNS disorders such as MS, NMOSD, autoimmune encephalitis, and narcolepsy type 1, HLA-shaped immune repertoires must be interpreted together with blood-brain or blood-CSF barrier access, local antigen distribution, cerebrospinal-fluid or intrathecal immune compartments, resident glial responses, and selective neuronal or glial vulnerability (9, 10, 23, 45, 55). These features can support recurrent or chronic inflammatory programs when peripheral immune priming, tissue access, antigen persistence, and local amplification interact over time.

Peripheral and neuromuscular disorders follow a different tissue logic. In GBS, post-infectious molecular mimicry can produce acute immune attack at peripheral nerves and roots through antibodies, complement, macrophages, and autoreactive T cells, whereas HLA associations remain heterogeneous (46–50). In MG, HLA effects are shaped by antibody subtype, thymic context, age at onset, and neuromuscular-junction biology rather than by a central glial program (27–30). Thus, HLA polymorphism provides a shared upstream immunogenetic filter, but central and peripheral neuroinflammatory programs diverge according to barrier architecture, target-cell biology, resident-cell participation, effector mechanism, and temporal course.

### 5.5 HLA association does not yet explain chronicity

The contribution of HLA variation to relapse, progression, and treatment response is less secure than its contribution to susceptibility. Persistent peptide presentation, epitope spreading, durable B-cell help, and local inflammatory feedback are plausible routes to chronicity (13, 58, 59). However, most available HLA studies are cross-sectional and were not designed to distinguish disease initiation from later trajectory.

Claims that HLA directly determines progression should therefore be avoided unless they are supported by replicated longitudinal analyses. The current evidence more safely supports HLA as one component of a broader system that includes environmental exposure, non-HLA immune genes, treatment, barrier state, and tissue-specific inflammatory amplification.

This boundary is clinically important. A strong susceptibility allele may have limited prognostic value, whereas a modest allele or haplotype may become informative when paired with a molecular subtype or immune phenotype.

### 5.6 Comparative evidence matrix

**Table 2** operationalizes the cross-disease synthesis by separating association strength from mechanistic evidence proximity. It shows how far the shared antigen-visibility principle can currently be followed in each disease, rather than assuming that all disorders have an equally resolved HLA-dependent pathway.

Association grades A, B, C, and U summarize replication and study design, whereas M1-M4 indicate the proximity of functional evidence to patient disease. Keeping these dimensions separate preserves the quantitative evidence upgrade without converting unlike forms of evidence into a single certainty score.

The matrix identifies MS as the strongest integrated mechanistic case, not necessarily the disease with the largest HLA effect. It also highlights where future work should prioritize peptide identification, receptor validation, ancestry replication, or longitudinal analysis.

## 6 Translational implications

### 6.1 Current clinical utility is selective and context dependent

HLA testing currently has selective adjunctive value rather than broad standalone utility across neuroimmune diseases. A genotype can strengthen biological interpretation in a defined setting, but it generally cannot establish diagnosis, predict outcome, or select therapy without clinical, serological, imaging, and ancestry information.

The clearest near-term uses are subtype-specific. DQB1*06:02 is highly enriched in narcolepsy type 1, and specific HLA class II haplotypes are enriched in anti-LGI1 encephalitis and MG subtypes (27–30, 32, 33, 54). Even in these settings, incomplete penetrance and population-dependent background frequencies preclude deterministic interpretation.

Clinical implementation should therefore be framed as an incremental-value question: whether HLA information improves discrimination, calibration, or clinical decision value beyond established variables in a specific subtype. Association alone is insufficient without a management consequence.

### 6.2 HLA is best integrated with immune endophenotypes

HLA information is most useful when paired with measured immune states. High-dimensional immune phenotyping provides a practical model: Gross and colleagues identified three blood immune endophenotypes in two early-MS cohorts that were associated with distinct inflammatory and structural disease trajectories (60). The study did not establish HLA-linked stratification, because HLA was not the defining analysis.

Its relevance is therefore methodological: phased HLA data could be tested prospectively together with cellular, proteomic, antibody, cerebrospinal-fluid, imaging, ancestry, and treatment-response measurements. In such models, HLA should be evaluated for added information beyond established clinical and molecular variables.

This integration keeps the translational claim bounded. HLA supplies an upstream immunogenetic variable, while immune endophenotypes and tissue markers capture the active disease process and its clinical consequences.

### 6.3 Precision neuroimmunology remains a research objective

HLA-informed precision neuroimmunology remains most credible as a research stratification strategy. High-resolution typing can improve cohort definition, expose ancestry-specific haplotypes, and generate testable hypotheses about peptide selection or effector bias, but it is not yet a routine treatment-selection tool across the disorders reviewed here.

Translation will require prospective multi-center cohorts, harmonized phenotype and serostatus definitions, ancestry-aware models, external validation, and direct comparison with simpler clinical predictors.

The appropriate sequence is mechanism discovery, replicated stratification, prospective validation, and only then clinical deployment. This sequence reduces the risk that strong genetic associations are prematurely converted into individual-level claims.

## 7 Current limitations and future directions

### 7.1 Causal inference is limited by LD, phenotype definition, and evidence type

Extensive LD across the MHC remains the central obstacle to causal interpretation. An associated classical allele may be causal, may act jointly with linked HLA allomorphs, or may tag regulatory or non-HLA variation. DR15 in MS and the 8.1 ancestral haplotype in MG illustrate why single-allele attribution requires conditional analyses and functional validation (23, 26, 27).

Phenotypic aggregation creates a second source of error. Historical NMO cohorts, broad MG categories, and mixed autoimmune encephalitis populations combine subgroups with different antibodies, ages at onset, tumor status, ancestries, and HLA effects (10, 27–30, 51, 53, 56). Future studies should define molecular subtype before estimating a genetic effect.

A third limitation is the frequent collapse of distinct evidence types. Association, binding, structural, ex vivo, model, and patient-tissue evidence answer different questions. Mechanistic chains should specify which links have direct support and which remain inferred. Association grades and mechanistic grades should therefore continue to be reported separately.

Progress depends less on accumulating additional isolated associations than on designing studies that connect a phased haplotype to peptide display, receptor recognition, measured immune state, and clinically relevant tissue biology.

### 7.2 Gene-environment interactions require disease-specific tests

Environmental exposure can modify an HLA-shaped immune repertoire, but the evidence is uneven. EBV-related antibody cross-reactivity, infected B-cell myelin peptide presentation, and DR15-restricted T-cell cross-recognition provide the most developed MS example (23, 24, 45). Smoking also interacts with DRB1*15 and absence of HLA-A*02 in replicated MS cohorts, although the biological route remains incompletely resolved (61).

Vitamin D has been linked experimentally to regulation of DRB1*15:01 expression, but this does not establish that vitamin D status explains the allele’s population-level effect (62). Microbiome-derived peptides are another plausible source of cross-reactivity, as illustrated by DR15-restricted recognition of selected microbial peptides (23). These observations should be treated as specific evidence, not as a universal HLA-environment mechanism.

Future interaction studies should be pre-specified, adequately powered, replicated across ancestries, and linked to functional assays. Exposure measurement must precede outcome when possible, and statistical interaction should not be presented as molecular interaction without experimental support.

### 7.3 Full-gene phasing and transplant resources can improve study design

Conventional low-resolution or exon-focused typing can miss intronic, regulatory, and phase information. The IPD-IMGT/HLA Database provides the reference nomenclature and sequence catalogue needed to interpret this diversity (16). Long-read platforms, including single-molecule real-time and nanopore sequencing, can recover full-gene or extended phased HLA sequences and reduce ambiguity (63, 64). The immediate research value is improved haplotype resolution and allele-specific functional annotation, not automatic clinical superiority.

Transplant immunology also offers methodological analogies and population-genetic resources, rather than direct models of autoimmune antigen recognition. Eplet-based matching represents HLA molecules as surface-exposed polymorphic configurations rather than allele names alone (65). Large donor registries, including the National Marrow Donor Program and Bone Marrow Donors Worldwide resources, provide population haplotype information and methods for ancestry-aware frequency estimation (66).

These frameworks should be transferred cautiously. Alloantibody immunogenicity and autoimmune peptide recognition are not equivalent biological problems. The most informative next studies will combine full-gene phasing, immunopeptidomics, TCR or antibody repertoire analysis, patient-derived functional assays, and longitudinal phenotyping in ancestrally diverse cohorts.

## 8 Conclusions

The principal conclusion of this Review is that HLA-dependent antigen visibility provides a shared upstream axis linking immunogenetic variation to neuroimmune disease, whereas its translation into effector responses and tissue injury is disease specific. MS, particularly the DR15 haplotype, is the most mechanistically resolved anchor for this model, connecting genetic association to the immunopeptidome, peptide-HLA-TCR recognition, cross-reactive immune repertoires, and patient-relevant functional evidence.

The cross-disease comparison shows why this anchor should not be generalized as a universal chain. Narcolepsy type 1 and anti-LGI1 encephalitis show large HLA-region effects despite unresolved initiating complexes; MG demonstrates subtype-dependent and sometimes opposing associations; NMOSD and anti-NMDAR encephalitis emphasize ancestry and molecular definition; and GBS shows that a strong mimicry mechanism can coexist with inconsistent HLA evidence. Central and peripheral neuroinflammatory programs therefore share an upstream antigen-visibility logic but diverge according to anatomical compartment, barrier structure, target-cell biology, effector mechanism, and temporal course.

HLA associations should therefore be interpreted within defined haplotypic, ancestral, molecular, and environmental contexts, with association strength kept distinct from mechanistic resolution. This evidence discipline does not replace the biological synthesis; it identifies where each link is established, suggestive, or unresolved. Phased full-gene typing, patient-derived antigen-receptor studies, multiethnic cohorts, and longitudinal immune phenotyping are the clearest routes toward mechanism-informed stratification. Routine individualized clinical use remains a future objective rather than a present capability.
